# Protective role of FBXL19 in *Streptococcus pneumoniae*-induced lung injury in pneumonia immature mice

**DOI:** 10.1186/s13019-023-02186-5

**Published:** 2023-03-24

**Authors:** Zhiqiang Chen, Bijuan Zheng, Zhiwei Zhang, Zhiyong Huang

**Affiliations:** Department of Neonatology, The Affiliated Hospital of Putian University, Putian, 351100 China

**Keywords:** Pneumonia, *Streptococcus pneumoniae*, Lung injury, Immature mice, FBXL19, FOMX1, Ubiquitination, Inflammation

## Abstract

**Objective:**

*Streptococcus pneumoniae* (*Spn*) is a common pathogen for pediatric pneumonia and leads to severe lung injury. This study is conducted to analyze the role of F-box and leucine rich repeat protein 19 (FBXL19) in *Spn*-induced lung injury in immature mice.

**Methods:**

Immature mice were infected with *Spn* to record the survival rates and bacterial loads in bronchoalveolar lavage fluid. Levels of FBXL19 and FOXM1 in lung tissues were determined via real-time quantitative polymerase chain reaction or Western blotting. After the interference of FBXL19, its impacts on lung inflammatory injury were appraised by the lung wet/dry weight ratio, myeloperoxidase activity, hematoxylin and eosin staining, and enzyme-linked immunosorbent assay. The binding of FBXL19 to forkhead box M1 (FOXM1) in mouse lung epithelial cells was determined. After MG132 treatment, the protein and ubiquitination levels of FOXM1 were measured. The functional rescue experiments were performed to analyze the role of FOXM1 in FBXL19-regulated lung injury.

**Results:**

FBXL19 was downregulated while FOXM1 was upregulated in lung tissues of *Spn-*infected immature mice. Overexpression of FBXL19 reduced the degree of lung injury and inflammation. FBXL19 can bind to FOXM1 to reduce its protein level via ubiquitination degradation. MG132 reduced the ubiquitination and increased the protein level of FOXM1. Overexpression of FOXM1 reversed the protective role of FBXL19 overexpression in lung injury of *Spn* immature mice.

**Conclusion:**

FBXL19 was downregulated by *Spn* and FBXL19 overexpression alleviated lung injury by inducing ubiquitination and degradation of FOXM1 in *Spn* immature mice.

**Supplementary Information:**

The online version contains supplementary material available at 10.1186/s13019-023-02186-5.

## Introduction

Pediatric pneumonia remains the major cause of childhood hospitalization and mortality, lacking optimal diagnostic methods [1]. *Streptococcus pneumoniae* (*Spn*) is one of the most common pathogens that colonize the nasopharynx and migrate to other parts of the airway and contributes to most cases of community-acquired pneumonia in childhood [2, 3]. *Spn* possesses the capacity to invade lung tissues and activate the epithelial response, destroying the alveolar capillary barrier [4]. However, the pathogenicity of *Spn* in lung injury is complex and is only investigated at the primary stage. Adding to the plight, the current treatment for bacterial pneumonia is often compromised by antibiotic resistance [5]. Therefore, it is vital to seek alternative therapies to alleviate *Spn*-induced lung injury and mortality.

Recently, the proteome analysis has revealed that an array of differentially expressed proteins are associated with *Spn*-induced lung injury [6]. Ubiquitination executed by three enzyme complexes (E1, E2, and E3) that link the ubiquitin chains to target proteins, plays a fundamental role in the homeostasis of intercellular proteins [7]. The Skp1-Cullin-1-F-box ligase complex is deemed as one of the largest ubiquitin E3 ligase families responsible for protein degradation [8]. F-box and leucine rich repeat protein 19 (FBXL19) is a newly identified member of F-box proteins [9] and is potent to mediate the ubiquitination and degradation of its target proteins [10–12]. Of note, FBXL19 plays a role in cancer progression [13, 14]. What’s more, FBXL19 is involved in inflammatory responses in rheumatoid arthritis [15]. More importantly, overexpression of FBXL19 mitigates apoptosis and inflammation in endotoxin-induced acute lung injury mice [16]. However, there is no relevant study on the role of FBXL19 in *Spn*-induced pneumonia.

On a separate note, forkhead box M1 (FOXM1), belonging to the FOX family of transcription factors, is a crucial player in pulmonary diseases [17]. Its dysregulation is prone to the occurrence of pulmonary fibrosis, pulmonary hypertension, and persistent lung inflammation [18–20]. Interestingly, FOXM1 is elevated in the serum of children with pneumonia and its knockdown retards lung injury and inflammation in vivo and in vitro [21]. Moreover, FOXM1 expression is altered by ubiquitination or deubiquitination [22, 23]. However, it is unclear whether FBXL19 mediates FOXM1 ubiquitination and further plays a role in *Spn*-induced pneumonia.

In light of the currently available literatures, we speculated that FBXL19 plays a regulatory role in lung injury in *Spn*-induced pneumonia immature mice via FOXM1, and aimed to confirm the role and downstream mechanism of FBXL19 in *Spn*-induced pneumonia immature mice in this study. In this manner, we hoped to provide novel targets for the treatment of pediatric pneumonia.

## Materials and methods

### *Spn*-induced pneumonia immature mice

All protocols for animal experiments were approved by the Ethics Committee of the affiliated hospital of Putian University (AF/SC-08/12/21.0) and the animal treatments complied with the Guide for the Care and Use of Laboratory Animals. Healthy BALB/c male mice (about 3 weeks, 12–15 g) were procured from Zhejiang Vital River Laboratory Animal Technology Co., Ltd. (Zhejiang, China, license No: SYXK(Zhejiang) 2019–0003) and housed in a continuously ventilated room with constant temperature of 20–25 °C under 12-h light/dark cycles and had ad libitum access to water and food. According to a previous study [24], a pneumonia immature mouse model was established through *Spn* induction. *Spn* (ATCC49619, American Type Culture Collection (ATCC), Rockville, MD, USA) was inoculated in THY sheep blood agar (Shanghai Aiyan Biotechnology Co., Ltd, Shanghai, China) overnight and cultured at 37 °C with 5% CO_2_ for 18 h. After centrifugation, bacteria were harvested and resuspended to 10^9^ colony-forming units (CFU)/mL in sterile phosphate-buffered saline (0.15 M, pH 7.2, PBS). Then, immature mice were anesthetized using an intraperitoneal injection of 50 mg/kg pentobarbital sodium (Sigma, St. Louis, MO, USA). Next, 100 μL of PBS containing 1 × 10^8^ CFU was injected into both nostrils of each immature mouse using the 29-gauge needle (50 μL/per nostril) to establish a pneumonia immature mouse model, named the SPN group. According to the prevention protocol, immature mice in the sham group were injected with sterile PBS. FBXL19 lentivirus overexpression vector, FOXM1 lentivirus overexpression vector, and their corresponding controls (Gemma Pharmaceutical Technology Co., Ltd, Shanghai, China) were intravenously injected into immature mice (the viral titer of 1 × 10^9^ TU/mL, the injection volume of 3 μL, viruses were dissolved in 100 μL PBS) 2 days before modeling [21]. After fixing the mice, the mouse tail was swabbed with 75% ethyl alcohol to disinfect and fill the blood vessels. The injection was performed at the site of 1/3 of the tail with the injection angel of 30° and using a disposable 1 mL insulin syringe (it is not easy to produce bubbles). After injection, the needle was retained for 15 min and then slowly pulled out to prevent the liquid from being taken out. The survival rates of immature mice within 14 days were recorded. After 14 days, all surviving immature mice were euthanatized using an intraperitoneal injection of 100 mg/kg pentobarbital sodium. Immature mice (N = 18) in each group were randomly subjected to measurement of the lung wet/dry weight ratio (N = 6), collection of broncho-alveolar lavage fluid (BALF) (N = 6), hematoxylin–eosin (H&E) staining, and analysis of tissue homogenate (N = 6).

### Lung wet/dry weight ratio

Water on the surface of fresh lung tissues was absorbed using filter papers. Then, the trachea and esophagus were removed by blunt dissection. Subsequently, the wet weight of the remaining lung tissues was measured using an electronic scale and recorded. Next, lung tissues were dried in an oven at 70 ℃ until the weight was constant to remove all moisture, and the dry weight was recorded. The wet/dry weight ratio was calculated as wet weight/dry weight [25]. Each experiment was performed in triplicate.

### Bacterial loads in BALF

After animal anesthesia, the chest was cut open to expose lung tissue. The trachea was incised open, intubated using a scalp needle and fixed with 0-gauge thread. Following that, lung tissues were separated, and the left main bronchus was ligated, and the right lung was flushed by an air tube. After endotracheal intubation, the right lung was slowly rinsed with 1 mL normal saline 3 times, and the lung tissue was gently massaged with the fingers. After 30 s, BALF in each group was collected and centrifuged at 4 °C and 260 g for 10 min. Precipitates were resuspended in 0.5 mL sterile PBS. The suspension was successively diluted into a tenfold diluent. The final diluent (50 mL) was coated on sheep blood agar and kept at 37 °C with 5% CO_2_. CFUs were counted after 18 h [24]. Each experiment was performed in triplicate.

### H&E staining

In brief, lung tissues in each group were fixed with 4% paraformaldehyde for 24 h, rinsed, dehydrated with gradient ethanol, and embedded in paraffin. Then, paraffined tissues were cut into 4 μm sections, dried in an incubator at 45 °C, and de-paraffined. Next, sections were stained with hematoxylin (Solarbio, Beijing, China) for 5 min, rinsed with running water for 3 s, differentiated with 1% hydrochloric acid ethanol for 3 s, and stained with 5% eosin (Solarbio) for 3 min. After dehydration, cleaning, and sealing, the morphology of lung tissues was observed under an optical microscope (Olympus, Tokyo, Japan) [21]. The tissues injury was scored by pathologists using the double-blind method with a 0–3 score system (0, normal; 1, mild; 2, moderate; 3, strong for interstitial/ alveolar edema, hemorrhage, alveolar septal thickening, and infiltration of the inflammatory cells [26]. Each experiment was performed in triplicate.

### Myeloperoxidase (MPO) activity

Lung tissues of immature mice were homogenized in Hank’s buffered salt solution. Lung tissues were fully rinsed with buffer to remove residual tissues and blood clots. Tissue shreds were poured into a homogenizer and the bottom of a homogenizing pipe was held in a large frozen beaker for 10 min and a pounder was used to fully grind to prepare the homogenate. Through centrifugation at 4℃ and 10,000 g for 20 min, the supernatant was obtained. Following the instructions provided by the manufacturer, MPO activity in lung homogenate was determined using an MPO activity assay kit (Colorimetric) (ab105136, Abcam, Cambridge, MA, USA) and a microplate reader (absorbance at 412 nm, Bio-Rad, Hercules, CA, USA) [24]. Each experiment was performed in triplicate.

### Enzyme-linked immunosorbent assay (ELISA)

Under the instructions of the producer, levels of interleukin (IL)-10 (ab255729, Abcam), IL-6 (ab222503, Abcam), and IL-1β (ab197742, Abcam) were determined using ELISA kits. The absorbance was determined using a microscope reader (Bio-Rad) [24]. Each experiment was performed in triplicate.

### Culture and treatment of lung epithelial cells

Mouse lung epithelial cells-12 (MLE-12, Shanghai Institute of Biochemistry and Cell Biology, Shanghai, China) were preserved in a Dulbecco's modified eagle medium/F-12 together with 10% fetal bovine serum and penicillin (100 U/mL)/streptomycin (100 μg/mL) and were cultured in moist air at 37 ℃ with 5% CO_2_. MLE-12 cells were infected with FBXL19 lentivirus overexpression vector and its negative control (multiplicity of infection = 20). After 48 h, stable cells were screened for the subsequent experiments [27].

### Co-immunoprecipitation (Co-IP) assay

To examine the binding of FBXL19 to FOXM1, lysates of the SPN mouse lung tissues and untreated MLE-12 cells were prepared using radioimmunoprecipitation assay (RIPA) buffer and incubated with antibodies anti-FBXL19 (ab172961, Abcam) and anti-IgG (ab133470, Abcam) at 4 ℃ overnight while slowly shaking. Then, incubated lysates were added with protein A/G beads and gently shaken at 4 ℃ for 2–4 h, so that antibodies were coupled with beads. After IP, lysates were centrifuged at 4 ℃ and 10,000* g* for 15 s to collect beads, and beads were washed to remove the non-specific binding. After elution using sodium dodecyl sulfate elution buffer, proteins were analyzed by Western blot assay [28]. Each experiment was performed in triplicate.

### Ubiquitination level determination

MLE-12 cells were treated with MG132 (10 μM) (MedChemExpress Co., Ltd., Monmouth Junction, NJ, USA) for 2 h, with dimethylsulfoxide (DMSO) (MedChemExpress Co., Ltd.) as the control [29]. Lung tissues or cells in different groups were lysed using RIPA buffer, heated at 100℃ for 10 min, and centrifuged at 12,000 g for 10 min. followed by determination of protein concentrations. Lysates were incubated with antibodies (FOXM1: ab207298, Abcam; IgG: ab133470, Abcam) (antibody/lysate = 1 μg/mg), followed by the addition of 30 μL protein A/G plus-agarose beads and overnight incubation at 4 ℃. Samples were added with 25–30 μL protein loading buffer and placed in metal bath at 100 ℃ for 5 min for degeneration. The ubiquitination level of FOXM1 was examined using an anti-Ubiquitin antibody (ab19247, Abcam). Each experiment was performed in triplicate.

### Real-time quantitative polymerase chain reaction (RT-qPCR)

The total RNA was separated using the TRIzol reagent (Invitrogen, Carlsbad, CA, USA). RNA was converted into the complementary DNA using a PrimeScript RT reagent kit (TaKaRa, Dalian, China). RT-qPCR was performed using SYBR Green qPCR SuperMix (Invitrogen) and ABI 7500 RT-PCR system (Applied Biosystems, Inc., Carlsbad, CA, USA). The PCR amplification program was as follows: 94 ℃ for 10 min, followed by 40 cycles at 94 ℃ for 15 s, 56 ℃ for 30 s, 72 ℃ for 1 min, and 72 ℃ for 10 min [21]. With GAPDH as the internal reference of FBXL19 and FOXM1, the relative expression amount was calculated with the help of the 2^−ΔΔCt^ method [30]. Primers used in RT-qPCR are shown in Table 1. Each experiment was performed in triplicate.

**Table 1:** PCR primers

Gene	Sequence (5′–3′)
FBXL19	F: GCCTGAAGATGGGAAAGGCT
	R: CTTGCTTGTCCGTCCTTCCT
FOXM1	F: TCAGCCAACCGTTCTCTCAC
	R: CAGGCCAGGGAAACTGATGT
GAPDH	F: GGTCCCAGCTTAGGTTCATCA
	R: AATCCGTTCACACCGACCTT

#### Western blot assay

The total protein was extracted using RIPA buffer (Beyotime, Shanghai, China), lysed at 4 ℃ for 15 min, and centrifuged at 15,000* g* for 15 min [21]. The protein was separated using sodium dodecyl sulfate–polyacrylamide gel electrophoresis gel and transferred onto polyvinylidene fluoride membranes. After blockade with 5% bovine serum albumin for 2 h, the membranes were incubated primary antibodies anti-FBXL19 (ab172961, 1:1000, Abcam) and anti-FOXM1 (ab207298, 1:1000, Abcam) at 4 ℃ overnight, with GAPDH (ab9485, 1:2500, Abcam) as the loading control. After washing with Tris-buffered saline with Tween 20, the membranes were incubated with secondary antibody (1:2000, ab205718, Abcam) for 1 h. Protein bands were visualized using a Gel Doc EZ Imager (Bio-Rad). Protein levels were quantified using the ChemiDoc XRS system (Bio-Rad) and the ratio of gray value of each protein to gray value of GAPDH was used for protein quantitative analysis. Each experiment was performed in triplicate.

#### Statistical analysis

All data were processed with SPSS21.0 statistical software (IBM Corp, Armonk, NY, USA) and GraphPad Prism 8.0 software (GraphPad Software Inc., San Diego, CA, USA) for statistical analysis and graphing. Data complied with normal distribution and homogeneity of variance. Mouse survival rates were analyzed using Kaplan–Meier plots and data among groups were analyzed by the log-rank test. Data between two groups were analyzed using the *t* test, and data among multiple groups were analyzed using one-way analysis of variance (ANOVA), followed by Tukey’s multiple comparison test. A value of *P* < 0.05 was indicative of statistical differences.

## Results

### ***Spn*** downregulates FBXL19 expression in lung tissues of pneumonia immature mice

**Fig. 1:**
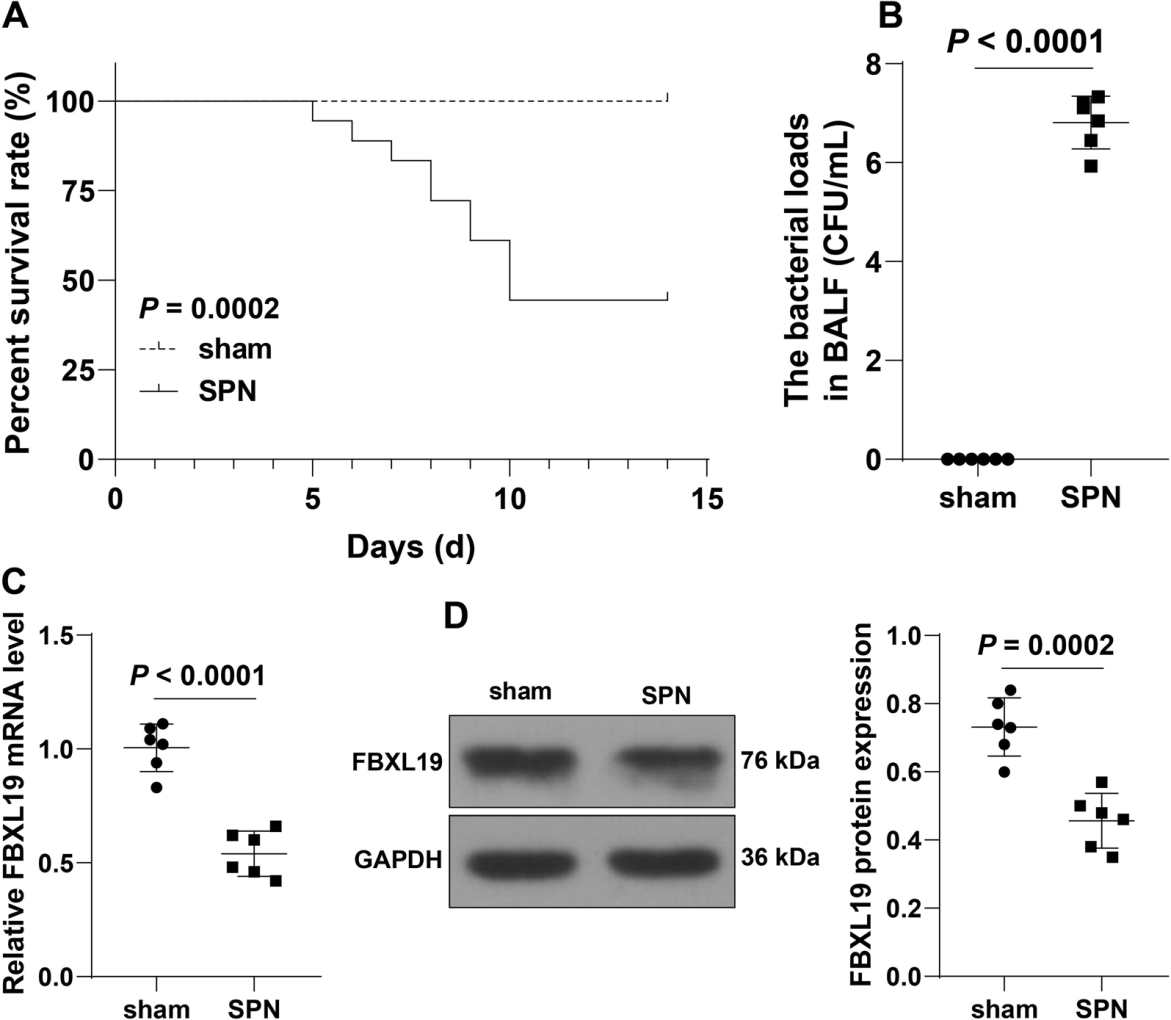
*Spn* downregulates FBXL19 expression in lung tissues of pneumonia immature mice. A pneumonia immature model (SPN) was established by *Spn* induction. **A** The survival rates of immature mice within 14 days were analyzed by Kaplan–Meier plots; **B** Bacterial loads in BALF in each group; **C**-**D** FBXL19 expression levels in lung tissues were determined by RT-qPCR (**C**) and Western blot assay (**D**). **A** N = 18; **B–D** N = 6. Experiments were repeated 3 times. Data in panels **B**, **C**, and **D** were analyzed using the *t* test, and data in panel **A** were analyzed by the log-rank test. BALF: broncho-alveolar lavage fluid

FBXL19 overexpression can attenuate endotoxin-induced acute lung injury [16]. But the role of FBXL19 in *Spn*-induced pneumonia remains unknown. It was observed that *Spn* treatment reduced the survival rates of immature mice (*P* = 0. 0002, Fig. 1A) and increased bacterial loads in BALF (*P* < 0. 0001, Fig. 1B). Meanwhile, we determined FBXL19 expression in lung tissues and found the downregulation of FBXL19 expression by *Spn* treatment (C: *P* < 0001, D: *P* = 0.0002, Fig. 1C-D).

### FBXL19 overexpression attenuates ***Spn***-induced lung injury in pneumonia immature mice

**Fig. 2:**
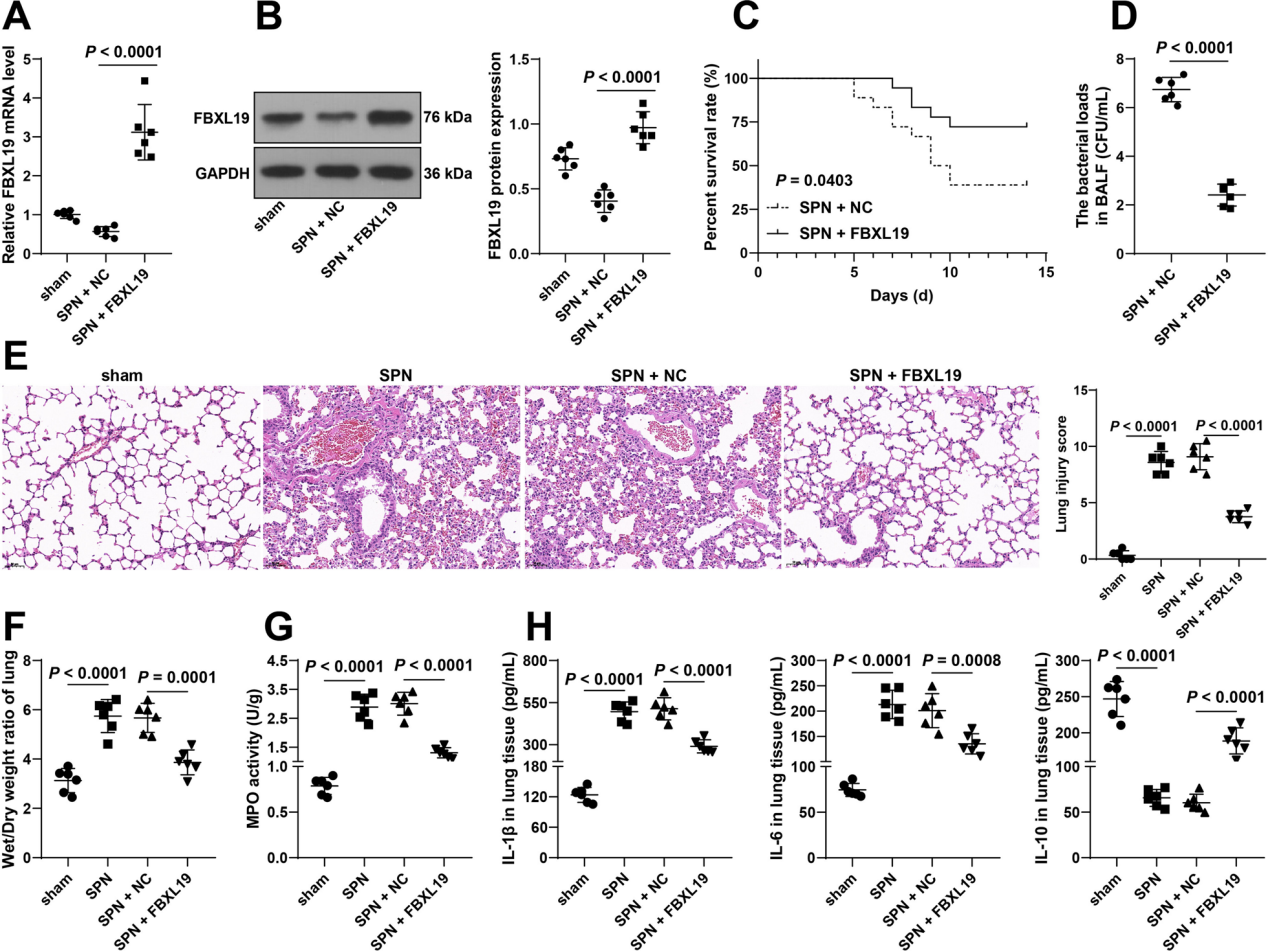
FBXL19 overexpression attenuates *Spn*-induced lung injury in pneumonia immature mice. Immature mice were intravenously injected with FBXL19 lentivirus overexpression vectors (FBXL19), with lentivirus empty vector (NC) as the negative control. Then, a pneumonia immature model (SPN) was established by *Spn* induction. **A**–**B**: FBXL19 expression levels in lung tissues were determined by RT-qPCR (**A**) and Western blot assay (**B**); **C** The survival rates of immature mice within 14 days were analyzed by Kaplan–Meier plots; **D** Bacterial loads in BALF in each group; **E** Lung injury scoring and representative images of H&E staining; **F** The lung wet/dry weight ratios; **G** MPO activity in lung tissues; **H** Contents of IL-1β, IL-6, and IL-10 in lung tissues examined by ELISA. **C** N = 18, **A**, **B** and **D–H** N = 6. Experiments were repeated 3 times. Data in panel **C** were analyzed using the log-rank test, data in panel **D** were analyzed by the* t* test, and data in panels **A**, **B**, **E**, **F**, **G**, and **H** were analyzed using one-way ANOVA, followed by Tukey’s multiple comparison test. *BALF* broncho-alveolar lavage fluid, *MPO* myeloperoxidase

To evaluate the impact of FBXL19 on lung injury, immature mice were treated with FBXL19 lentivirus overexpression vectors, resulting in successful upregulation of FBXL19 expression in lung tissues (Both *P* < 0.0001, Fig. 2A-B). After FBXL19 overexpression, the survival rates of immature mice were increased (*P* = 0.0403, Fig. 2C) and bacterial loads in BALF were decreased (*P* < 0.0001, Fig. 2D). In addition, *Spn* treatment enhanced the injury of lung tissue, while FBXL19 overexpression lessened the lung injury (*P* < 0.0001, Fig. 2E); *Spn* treatment augmented the lung wet/dry weight ratios and MPO activity (*P* < 0.0001, Fig. 2F-G), while FBXL19 overexpression declined the lung wet/dry weight ratio (*P* = 0.0001, Fig. 2F) and MPO activity (*P* < 0.0001, Fig. 2G); *Spn* treatment brought about increases in contents of IL-1β and IL-6 and a decrease in IL-10 content (Both *P* < 0.0001, Fig. 2H), while FBXL19 overexpression reversed these changes (IL-1β and IL-10: *P* < 0.0001, IL-6: *P* = 0.0008, Fig. 2H). Overall, FBXL19 overexpression attenuated *Spn*-induced lung injury in pneumonia immature mice.

### FBXL19 binds to FOXM1 and inhibits its protein level

**Fig. 3:**
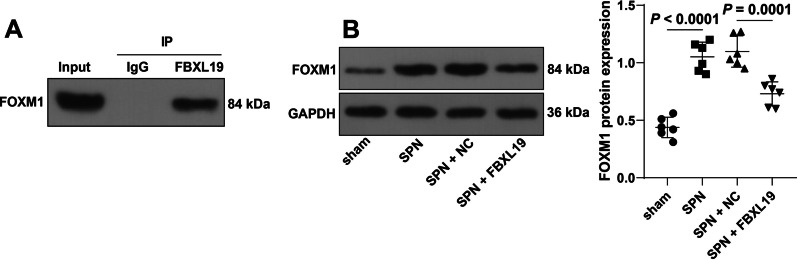
FBXL19 binds to FOXM1 and inhibits FOXM1 protein level. **A** The binding of FBXL19 to FOXM1 in SPN mouse lung tissue was tested by the Co-IP assay; **B** Protein levels of FOXM1 in lung tissues were measured by Western blot assay, N = 6. Experiments were repeated 3 times. Data in panel **B** were analyzed using one-way ANOVA, followed by Tukey’s multiple comparison test

FBXL19 as a ubiquitin ligase, can catalyze ubiquitination modification and induce protein degradation [10]. FOXM1 can be modified by ubiquitination [31] and is upregulated in the serum of pneumonia patients [21]. We speculated that FOXM1 upregulation is associated with FBXL19. First, the Co-IP assay revealed a binding relationship between FBXL19 and FOXM1 in the SPN mouse lung tissue (Fig. 3A). The protein levels of FOXM1 were elevated in lung tissues of *Spn* mice (*P* < 0.0001, Fig. 3B) but decreased in lung tissues with overexpression of FBXL19 (*P* = 0.0001, Fig. 3B). Overall, FBXL19 bound to FOXM1 and inhibited its protein level.

### FBXL19 ubiquitinates and degrades FOXM1

**Fig. 4:**
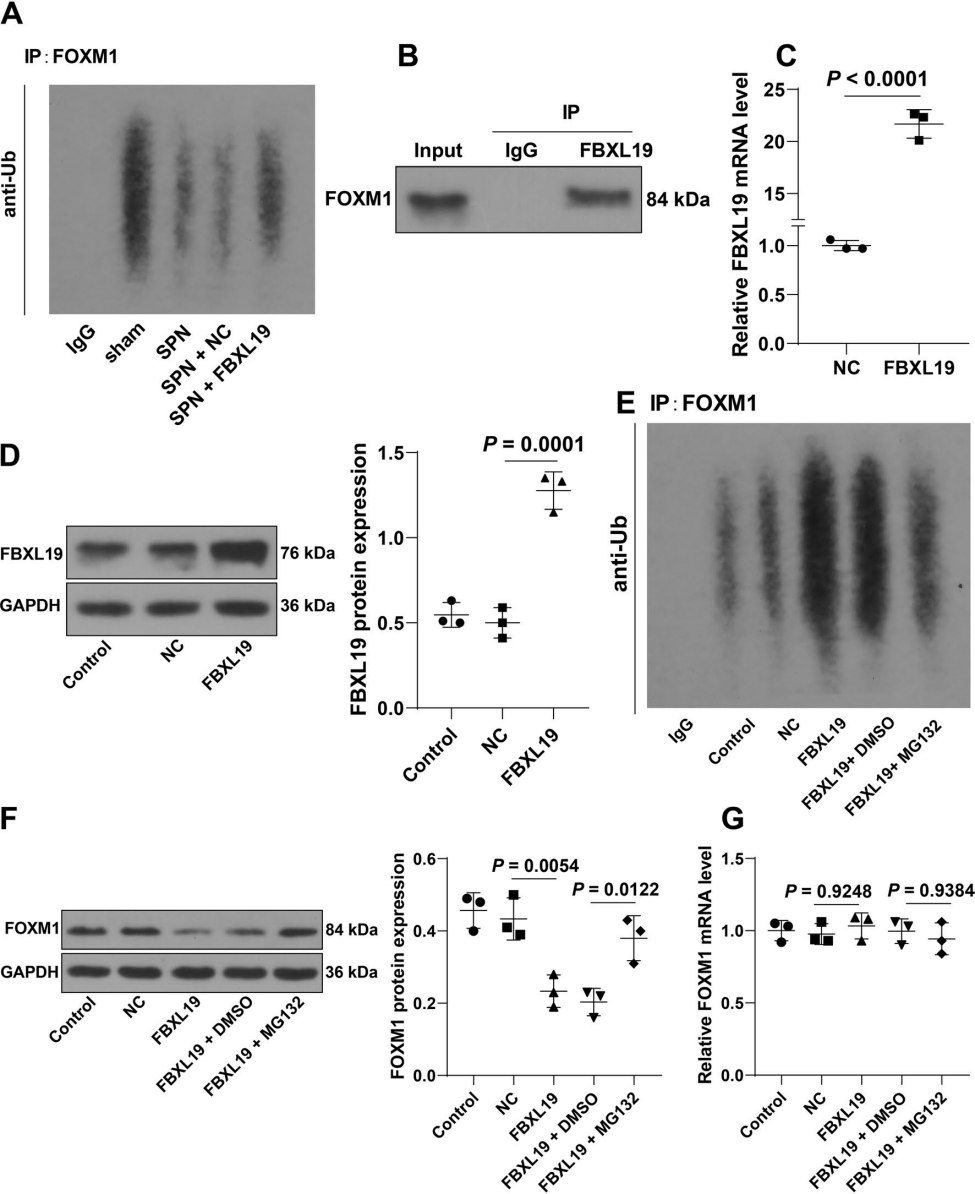
FBXL19 ubiquitinates and degrades FOXM1. **A** Ubiquitination levels of FOXM1 in lung tissues; **B**: The binding of FBXL19 to FOXM1 was tested by the Co-IP assay; MLE-12 cells were infected with FBXL19 lentivirus overexpression vectors (FBXL19), with lentivirus empty vector (NC) as the negative control; **C**, **D** FBXL19 expression levels in cells were determined by RT-qPCR (**C**) and Western blot assay (**D**); MLE-12 cells were treated with MG132, with dimethylsulfoxide (DMSO) as the negative control; **E **Ubiquitination levels of FOXM1 in cells; **F**–**G** FOXM1 expression levels in cells were determined by Western blot assay (**F**) and RT-qPCR (**G**). Experiments were performed in triplicate. Data in panel **C** were analyzed using the *t* test, and data in panels **D**, **F**, and **G** were analyzed using one-way ANOVA, followed by Tukey’s multiple comparison test

Next, to confirm that FBXL19 regulates FOXM1 protein level via ubiquitination modification, we measured the ubiquitination levels of FOXM1 in lung tissues and found that *Spn* treatment decreased the ubiquitination levels of FOXM1 in lung tissues, while FBXL19 overexpression augmented the ubiquitination levels of FOXM1 (Fig. 4a). Then, MLE-12 cells were cultured, and the binding of FBXL19 to FOXM1 was found in cells (Fig. 4b). After MLE-12 cells were infected with FBXL19 lentivirus overexpression vectors to upregulate intercellular expression of FBXL19 (C: *P* < 0.0001, D: *P* = 0.0001, Fig. 4c, d), the ubiquitination levels of FOXM1 were increased, and the protein levels of FOXM1 were significantly declined (F: *P* = 0.0054, Fig. 4e, f), but the mRNA levels of FOXM1 had no change (*P* = 0.9248, Fig. 4g). Cells were treated with the proteasome inhibitor MG132 to downregulate the intercellular ubiquitination levels, upon which the protein levels of FOXM1 were elevated accordingly (*P* = 0.0122, Fig. 4e), but the mRNA levels of FOXM1 had no change (*P* = 0.9384, Fig. 4g). Overall, FBXL19 degraded FOXM1 via ubiquitination.

### FOXM1 overexpression counteracts the protective role of FBXL19 overexpression in lung injury in pneumonia immature mice

**Fig. 5:**
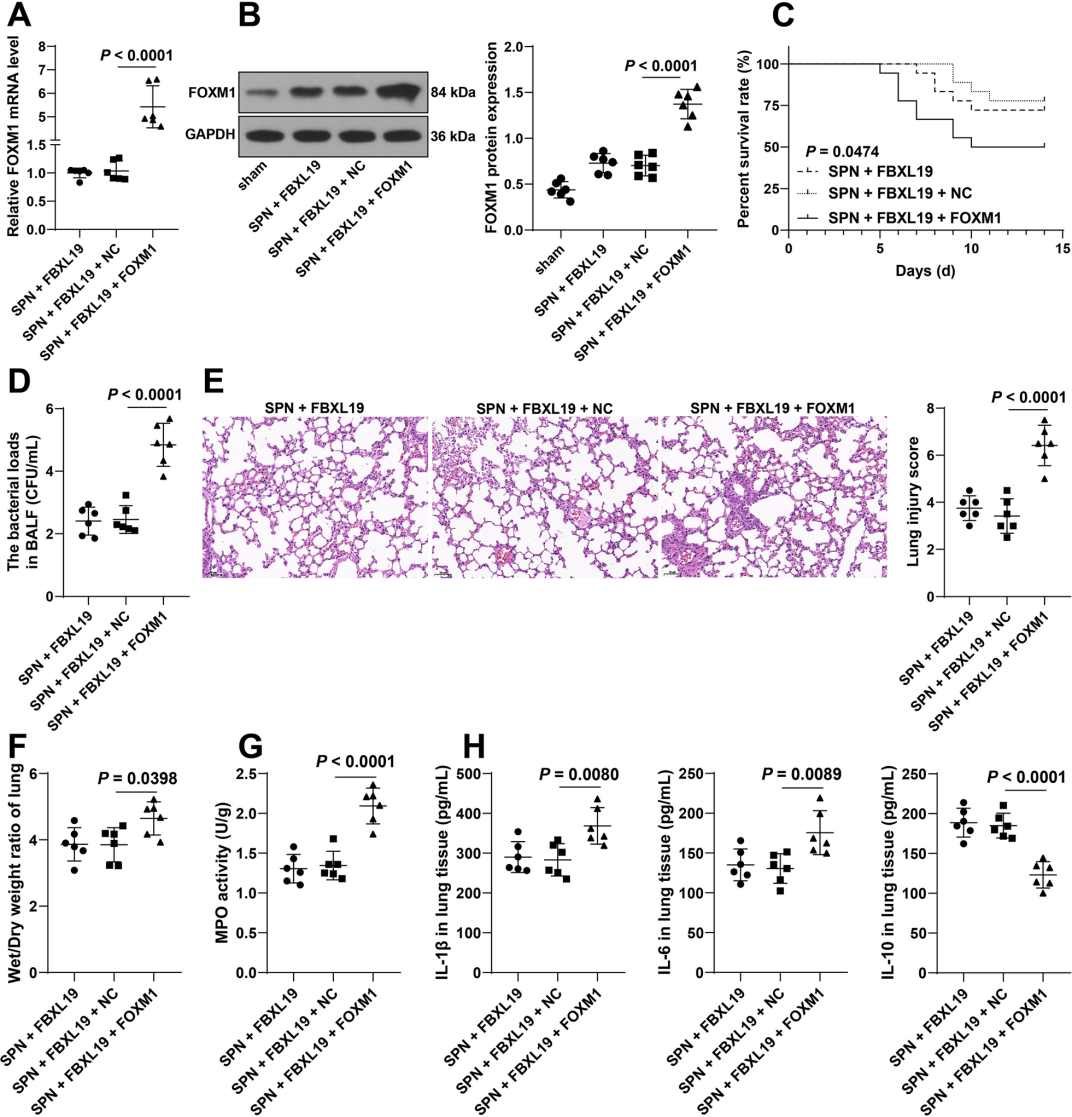
FOXM1 overexpression counteracts the protective role of FBXL19 overexpression in lung injury in pneumonia immature mice. Immature mice were intravenously injected with FOXM1 lentivirus overexpression vectors (FOXM1), with lentivirus empty vector as the negative control (NC). Then, a pneumonia immature model (SPN) was established by *Spn* induction. **A**, **B** FOXM1 expression levels in lung tissues were determined by RT-qPCR (**A**) and Western blot assay (**B**); **C**: The survival rates of immature mice within 14 days were analyzed by Kaplan–Meier plots; **D** Bacterial loads in BALF in each group; **E**: Lung injury scoring and representative images of H&E staining; **F** The lung wet/dry weight ratios; **G**: MPO activity in lung tissues; **H** Contents of IL-1β, IL-6, and IL-10 in lung tissues examined by ELISA. **C** N = 18, **A**, **B** and **D–H** N = 6. Experiments were repeated 3 times. Data in panel **C** were analyzed by the log-rank test, and data in panels **A**, **B**, **D**, **E**, **F**, **G**, and **H** were analyzed using one-way ANOVA, followed by Tukey’s multiple comparison test. BALF: broncho-alveolar lavage fluid, MPO: myeloperoxidase

Finally, immature mice were treated with FBXL19 lentivirus overexpression vectors and FOXM1 lentivirus overexpression vectors, resulting in successful upregulation of FOXM1 in lung tissues (Both *P* < 0.0001, Fig. 5a, b). After FOXM1 overexpression, the survival rates of immature mice were decreased (*P* = 0.0474, Fig. 5c) and bacterial loads in BALF were increased (*P* < 0.0001, Fig. 5d), the degree of lung injury was elevated (*P* < 0.0001, Fig. 5e) and the lung wet/dry weight ratios and MPO activity were augmented (F: *P* = 0.0398, G: *P* < 0.0001, Fig. 5f, g), and the contents of IL-1β and IL-6 were upregulated and IL-10 content was downregulated in lung tissues (IL-1β: *P* = 0.0080, IL-6: *P* = 0.0089, IL-10: *P* < 0.0001, Fig. 5h). Overall, FOXM1 overexpression counteracted the protective role of FBXL19 overexpression in lung injury in pneumonia immature mice.

## Discussion

Pediatric pneumonia is a common childhood disorder requiring hospitalization and high-cost treatment [32]. *Spn* is a common pathogen for pediatric pneumonia and results in pathological changes in the lung [3, 24]. Understanding the regulation of differentially expressed proteins in *Spn*-induced pneumonia is conducive to the identification of diagnostic biomarkers and therapeutic targets. Herein, we uncovered FBXL19 and FOXM1 as regulators of lung injury in *Spn*-induced pneumonia immature mice and validated a mechanism wherein FBXL19 bound to FOXM1 and induced the ubiquitination and degradation of FOXM1, thus attenuating lung injury in *Spn*-induced pneumonia immature mice.

The combination of F-box proteins can control the tumor necrosis factor receptor-associated factor adaptor stability to fine-tune cytokine driven-inflammation where the F-box and leucine-rich repeat protein inhibits cytokine secretion [33]. A prior study has reported that FBXL19 prevents sepsis-induced lung injury by degrading the IL-33-ST2L axis [16]. MPO, a neutrophil enzyme, is correlated with oxidative stress and morbidity in lung inflammation [34]. Besides, IL-1β and IL-6 play a pro-inflammatory role in childhood community-acquired pneumonia while IL-10 counteracts inflammatory responses [35]. In this study, *Spn* treatment downregulated the expression levels of FBXL19, together with decreased survival rates of immature mice, increased bacterial loads in BALF, inflammatory infiltration, and elevated lung wet/dry weight ratio and MPO activity, whereas overexpression of FBXL19 averted the above outcomes. Consistently, recruitment of FBXL19 inhibits lipopolysaccharide (LPS)-induced H4K8 acetylation of the cytokine, thus lessening cytokine release in lung epithelial cells [36]. Likewise, FBXL19 is one of the hub genes in rheumatoid arthritis that exert anti-inflammatory functions [15]. Collectively, our findings and a plethora of evidence made it plausible that FBXL19 overexpression mitigates *Spn*-induced lung injury in pneumonia immature mice.

FBXL19 possesses the capacity of degrading target proteins via ubiquitination [10–12]. Increased FOXM1 levels have been observed in lung tissues of patients with newborn hyperoxic lung injury and the serum of patients with childhood pneumonia [21, 37], which is in accordance with the upregulation of FOXM1 in *Spn*-induced immature mice in our study. In addition, FOXM1 has experienced ubiquitination and deubiquitination mediated by ubiquitin E3 ligases and deubiquitinases in diseases and cancers [22, 23]. Our results revealed that FBXL19 binding to FOXM1 decreased the protein levels of FOXM1 and increased the ubiquitination levels of FOXM1 but induced no change in FOXM1 mRNA level. Furthermore, overexpression of FOXM1 reversed the protective role of FBXL19 overexpression against lung injury in pneumonia immature mice. Specifically, FOXM1 can affect inflammation in pneumonia through NF-κB and the JAK/STAT signaling pathway [38] and FOXM1 deficiency reduces the expressions of CCL11, CCL24 and chemokine receptors CCR2 and CX3CR1 to further affect inflammation [39]. These evidences indicate that there are many downstream mechanisms of FOXM1 to affect inflammatory responses and it requires more experiments to validate the downstream mechanism of FOXM1 in *Spn*-induced lung injury. Moreover, following inflammatory lung injury, FOXM1 can promote endothelial and vascular repair and improve lung vascular permeability and lung functions, hinting at the reparative role of FOXM1 [37, 40, 41]. For this reason, we conjectured the biphasic pattern of FOXM1 in pneumonia with an initial adverse role in initiating inflammatory injury and the subsequent role in promoting the recovery and survival after the disease. Nevertheless, future studies are needed to provide compelling evidence for the biphasic pattern of FOXM1 in pneumonia. Altogether, our findings initially demonstrated that FBXL19 protectes mice from *Spn*-induced lung injury by mediating the ubiquitination and degradation of FOXM1.

## Conclusions

In summary, our study for the first time demonstrated the protective role of FBXL19 in *Spn*-induced pneumonia immature mice via ubiquitination and degradation of FOXM1, which provides a theoretical reference for the clinical study of FBXL19 in pediatric pneumonia. However, our study only confirmed a single molecular mechanism of FBXL19 in *Spn*-induced pneumonia immature mice, lacking exploration of the mechanism of FBXL19 and FOXM1 downregulation. In addition, the change of FOXM1 mRNA level in the lung and FOXM1 downstream mechanism in *Spn*-induced pneumonia remain unknown. Moreover, we failed to validate our mechanism in multiple cell types or determine which type of pneumonocytes has the highest expression of FBXL19 and FOXM1. As the intravenous injection of lentiviruses might affect the rest of the body, whether the change of FBXL19 in other organs can affect lung injury is unknown. In the future, more studies are essential to validate the role of FBXL19 in other aspects of pneumonia in animals, such as pyroptosis, and explore FBXL19 upstream mechanism and FOXM1 downstream mechanism, and confirm the role of the FBXL19/FOXM1 axis in other infection/lung injury models and other types of cells and through other injection methods, such as nasal injection, seeking novel targets for the treatment of pneumonia.

## Supplementary Information

**Additional file 1.** Unedited Western blot membranes.

## Data Availability

The datasets used and/or analysed during the current study are available from the corresponding author on reasonable request.
