# Supplementary material for: Protective role of FBXL19 in Streptococcus pneumoniae-induced lung injury in pneumonia immature mice
Source: J Cardiothorac Surg. 2023 Mar 24;18:92. doi: 10.1186/s13019-023-02186-5 (PMC10037874; doi:10.1186/s13019-023-02186-5)
Supplement: Supplementary file 1 — Additional file 1. Unedited Western blot membranes. [file 13019_2023_2186_MOESM1_ESM.pdf]

Figure 1D

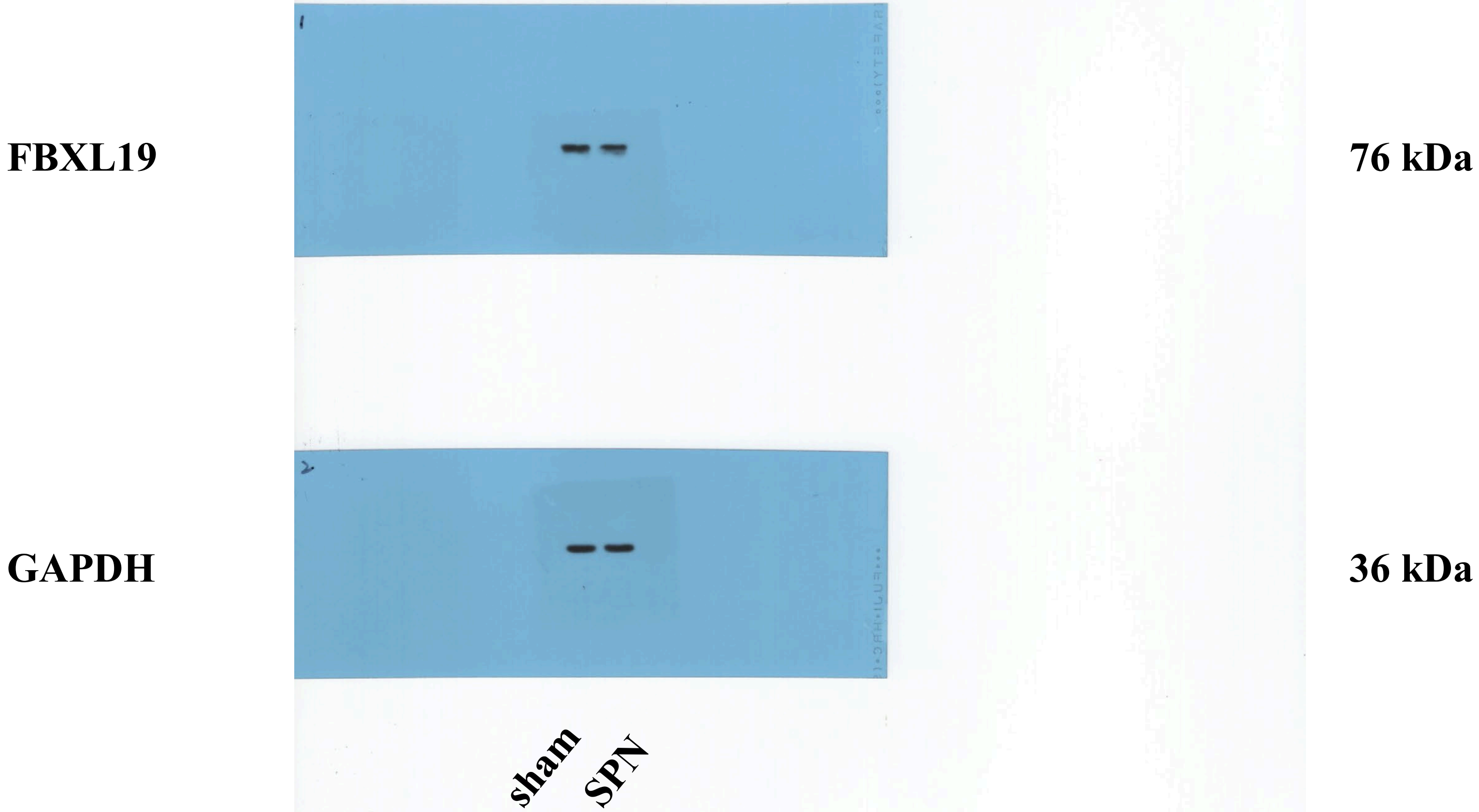

Figure 2B

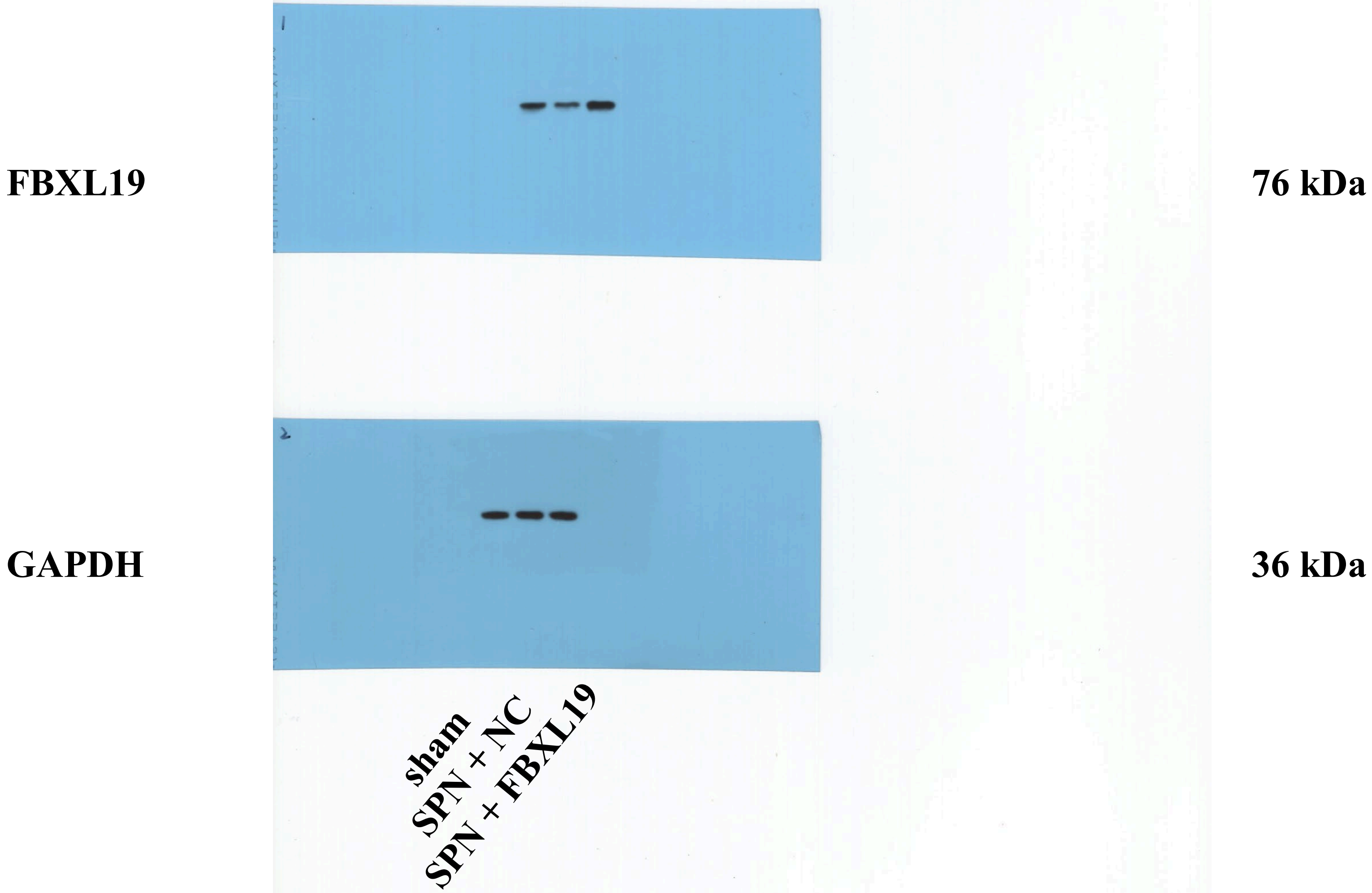

Figure 3A

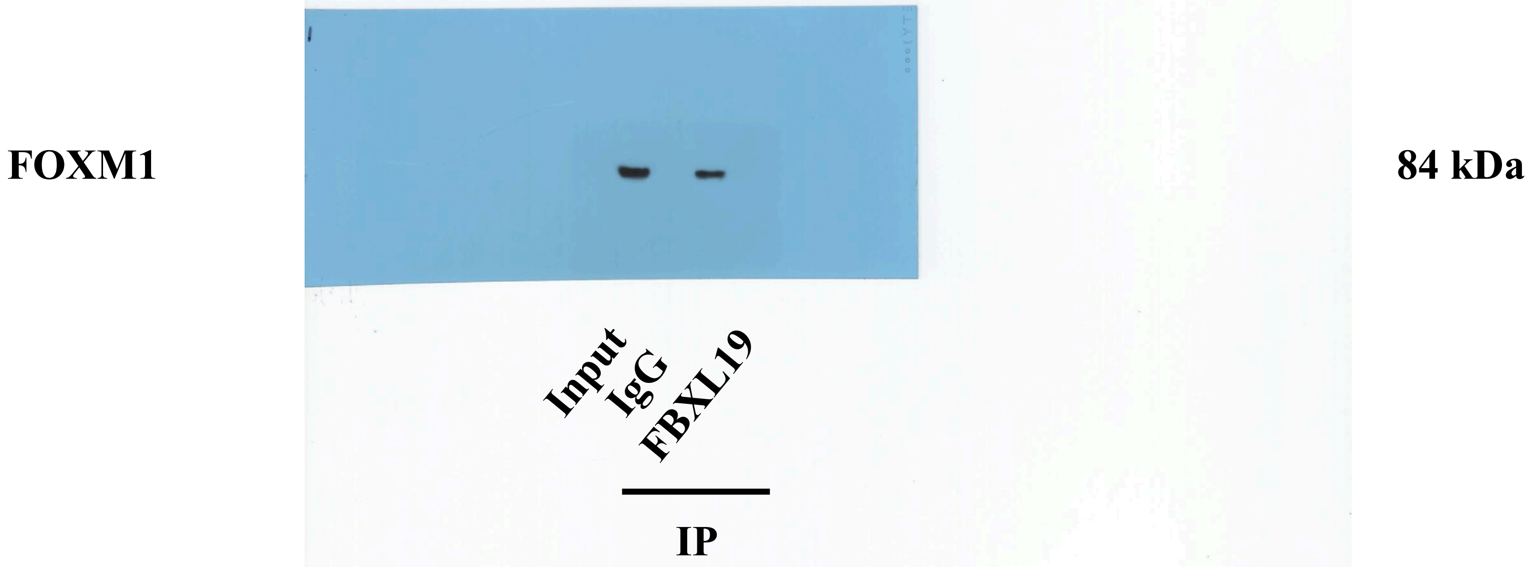

Figure 3B

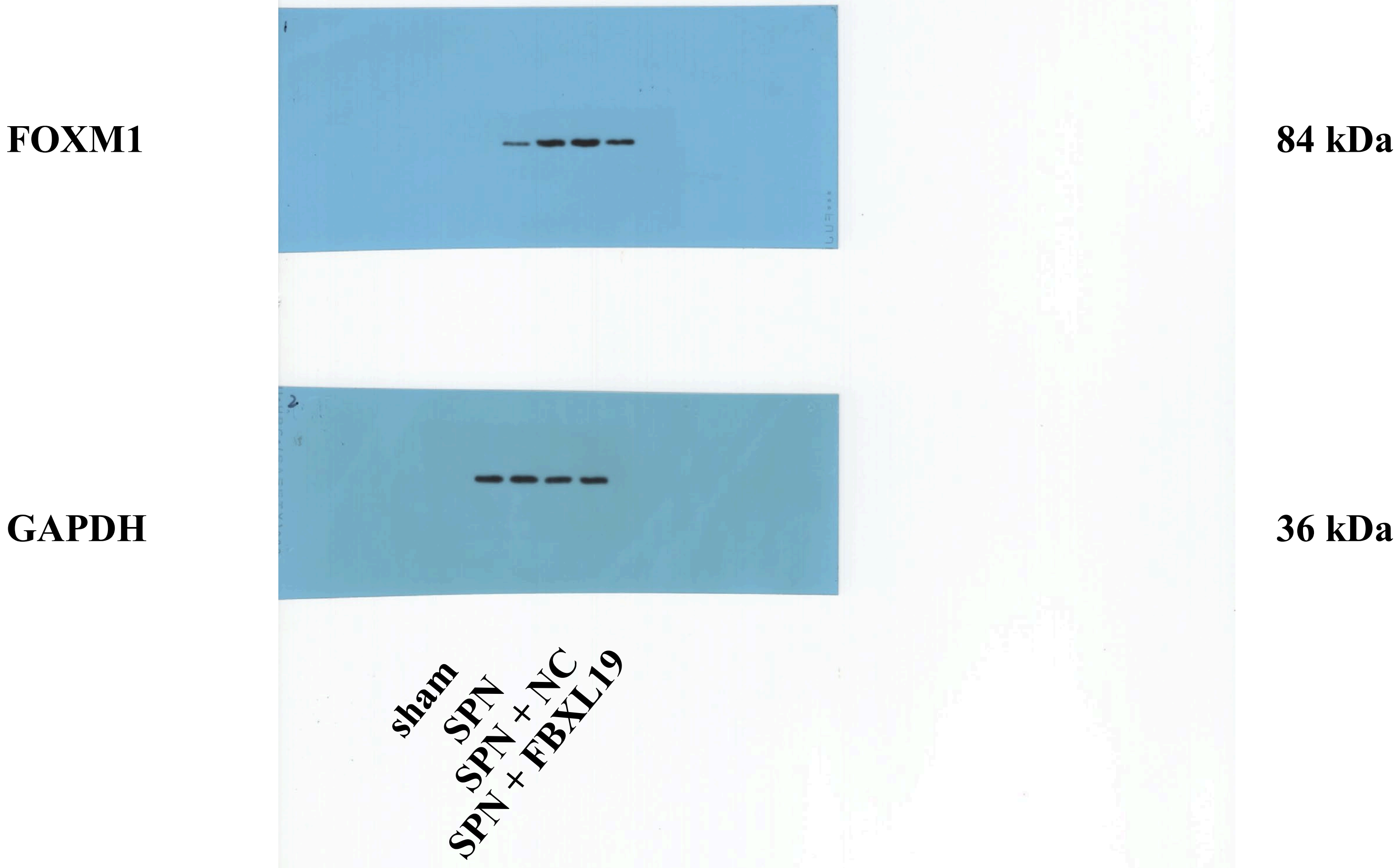

Figure 4A and 4E

IP: FOXM1

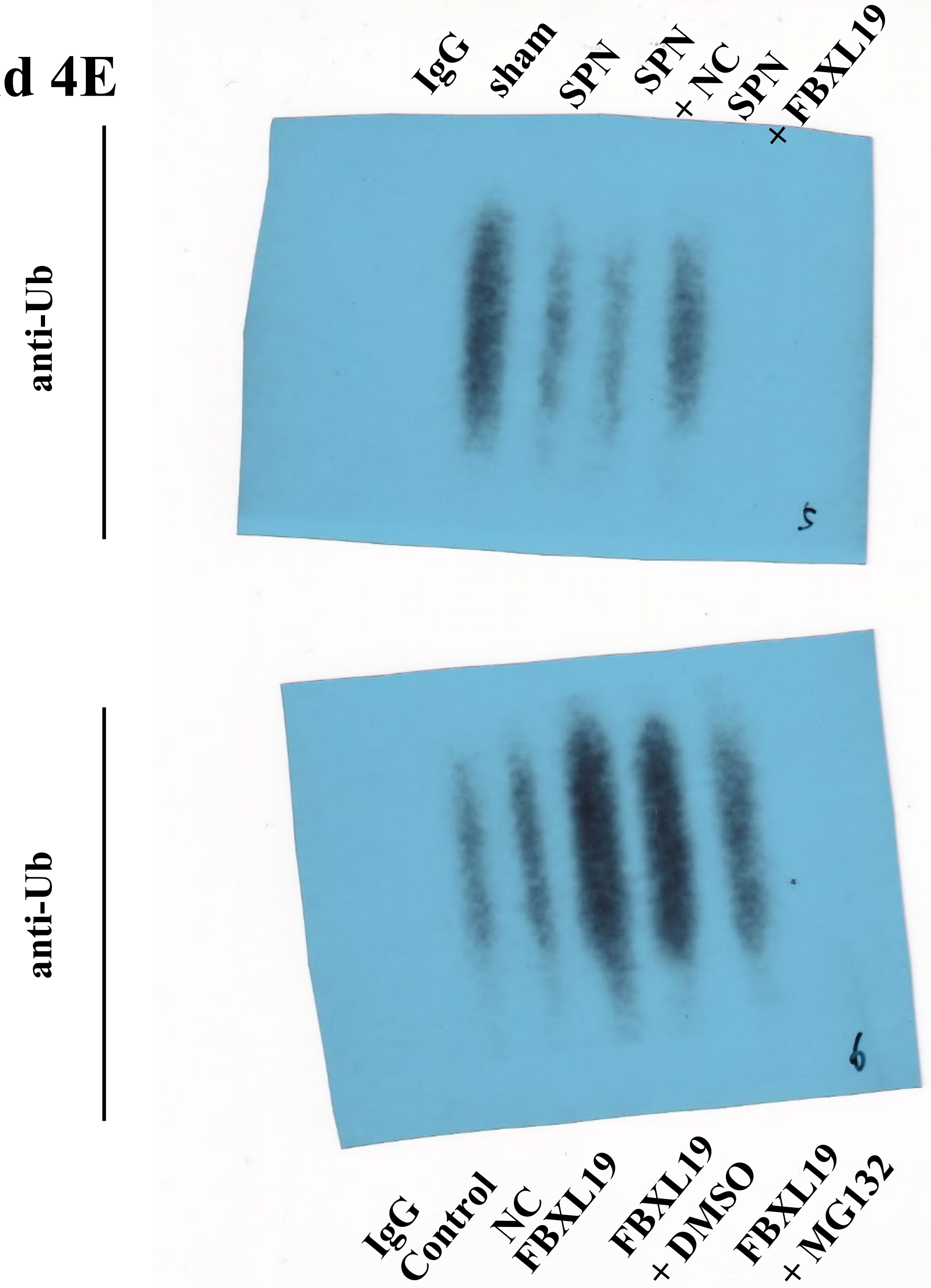

Figure 4B

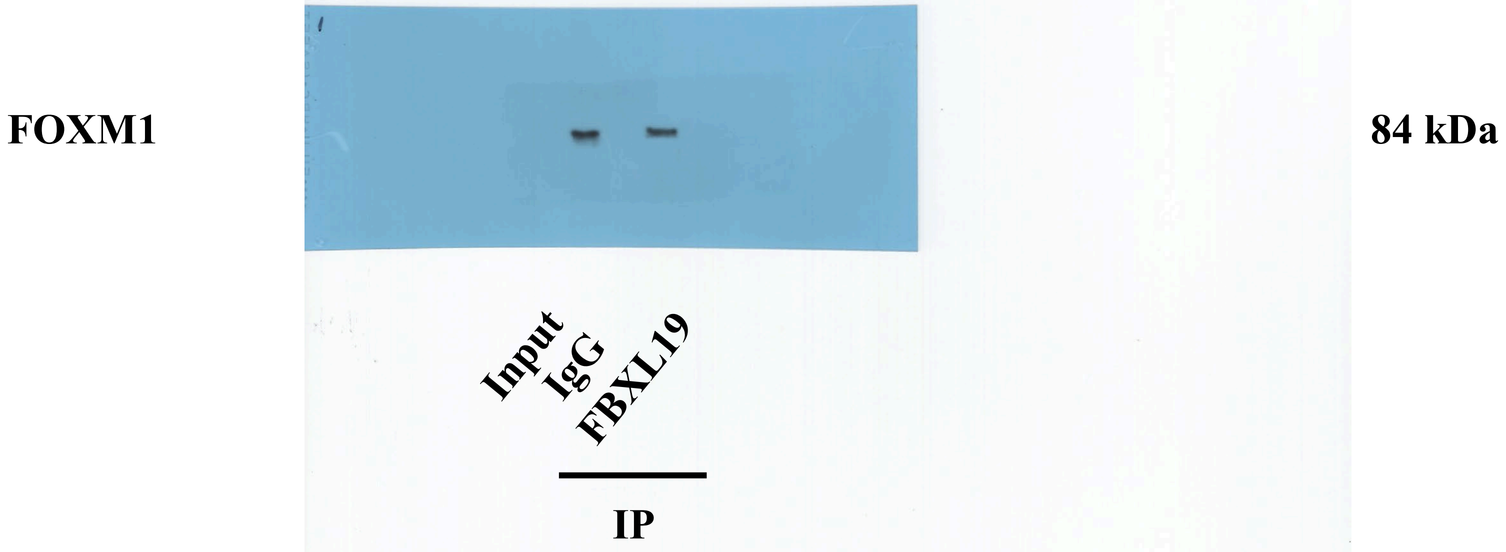

Figure 4D

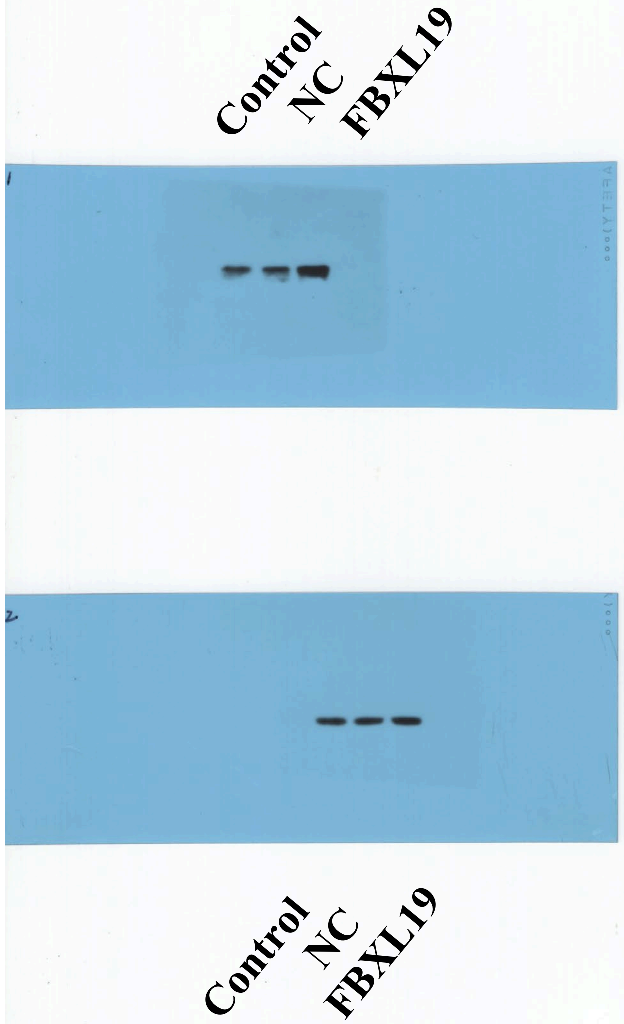
Control
NC
FBXL19
FBXL19
76 kDa
GAPDH
36 kDa
Control
NC
FBXL19

Figure 4F

FOXM1 84 kDa

GAPDH 36 kDa

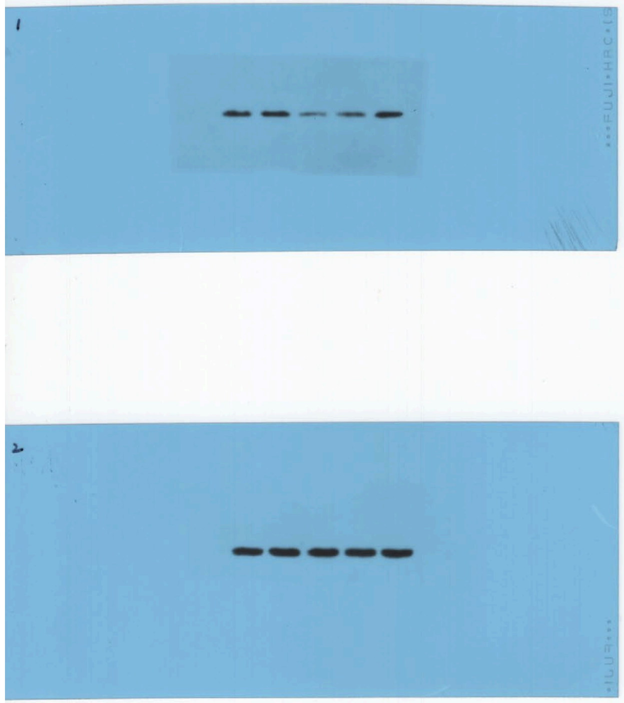

Control
NC
FBXL19
FBXL19 + DMSO
FBXL19 + MG132

Figure 5B

FOXM1

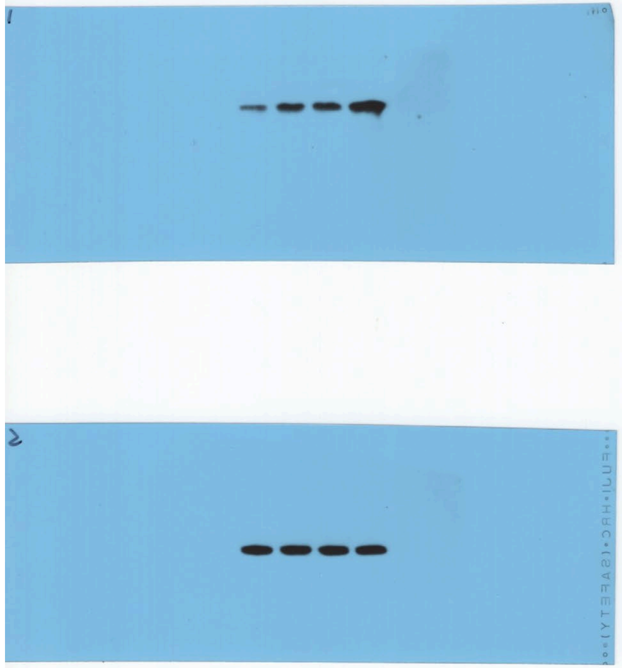

84 kDa

GAPDH

36 kDa

sham

SPN + FBXL19

SPN + FBXL19 + NC

SPN + FBXL19 + FOXM1
